# The role of leadership in collective creativity and innovation: Examining academic research and development environments

**DOI:** 10.3389/fpsyg.2022.1060412

**Published:** 2022-12-22

**Authors:** Zijian Huang, Stavros Sindakis, Sakshi Aggarwal, Ludivine Thomas

**Affiliations:** ^1^School of Economics, Yunnan University, Kunming, China; ^2^School of Social Sciences, Hellenic Open University, Patras, Greece; ^3^National Research Base of Intelligent Manufacturing Service, Chongqing Technology and Business University, Chongqing, China; ^4^Institute of Strategy, Entrepreneurship and Education for Growth (iSEEG), Paphos, Cyprus; ^5^Business and Law University of Roehampton London, Roehampton, United Kingdom

**Keywords:** leadership role, transformational leadership, LMX theory, ambidextrous leadership, creativity and innovation, research and development

## Abstract

**Introduction:**

Leadership is pragmatically linked to innovation adoption and implementation at a team level, as managers oversee the strategic decisions and policymaking, control resources, and moderate the scanning and searching of the environment. The paper attempts to provide new concepts and examines theoretical and practical implications to better understand how the leadership role is executed in an R&D environment to foster team creativity and innovation.

**Methods:**

A quantitative analysis was plausible over qualitative research mainly because the survey was conducted using a single technique, employing a questionnaire that was selected after checking the principal component analysis (PCA) and confirmatory factor analysis (CFA).

**Results:**

The findings show that the production of ideas positively impacts leadership, leading to growth and competitive advantage for the organization. Also, the PMEG (people, means, effects, goals) framework will positively impact leadership as leaders focus on those factorsthat influence an individual’s attitudes, behaviors, and interactions between groups.

**Discussion:**

The paper highlights the involvement of R&D organizations and groups in developing innovative products, services, technologies, and processes that further positively impact a team. This study is the first to highlight the role of the PMEG framework with the factors that influence an individual’s attitudes, behaviors, and interactions between groups. The study’s main contribution is to explore creativity as a potential mediator for leadership–organizational innovation.

## Introduction

Research and development depend significantly on creative minds that will be able to fashion tomorrow’s innovations. Barsh et al. (2008) explored that innovation is predicted to become one of the primary drivers of growth in the coming years. Previous research (e.g., Amabile, 2012; Kesting et al., 2015; Yi et al., 2017) has shown that the majority of innovative ideas (about 80%) are implemented by employees, who are a vital source of innovation. Innovative employee ideas give firms the ability to achieve their objectives and grow. For instance, Shafique et al. (2019) investigated the patterns of connections between a transformational leadership style and organizational-level innovation *via* individual-level creativity. In a different study, Shafique et al. (2019) used a multi-level model to examine the impact of authentic leadership on team creativity *via* individual-level creativity.

Further, Amabile and Pratt (2016) cited a model of organizational innovation incorporating individual creativity. This model reveals the concepts of organizational innovation and individual creativity. Moreover, the corporate environment impacts individual creativity, while individual creativity reinforces organizational innovation. In addition to building up a favorable climate for creativity, leaders’ roles are to ensure their subordinates remain actively involved in their work and try to generate innovative products, techniques, and methods to remain competitive (Shalley and Gilson, 2004). This holds true for scientists in an R&D environment, who are particularly interested in motives such as intellectual challenges or autonomy (Sauermann and Cohen, 2010). Therefore, Rosso (2014) claims that the componential model of creativity influences organizational creativity, which suggests that motivation is the drive to engage in exciting and essential creative outputs.

The componential theory of creativity emphasized by Amabile (1988) through the mediational model proposes that a leader’s behavior influences subordinate perceptions of leader support that, in turn, influence creativity and is the aspect that is the most directly influenced by the supervisor (Amabile et al., 2004). Further work revealed that interactions between leaders and subordinates could influence “perception, feelings and performance” and can, over time, positively inspire creativity by fostering subordinates’ intrinsic motivation (Amabile et al., 2004; Amabile, 2012). The study also revealed that leaders who did keep team members informed about stressful issues recognized exemplary performance in public or reacted to problems in work with understanding and helped correlated positively with apparent support. They, however, pointed to the need for additional studies to further examine these characteristics and, of interest, the relationship between creativity, innovation, and leadership style. Thus far, the authors suggest the need to explore these characteristics further to gain further insights into the roles they play, for example, in the R&D environment. The present study will thus attempt to determine the leader’s characteristics that enhance subordinates’ motivation towards creativity and environment in the academic and R&D-based environment.

## Theoretical background

### Leadership outline

**Figure 1:**
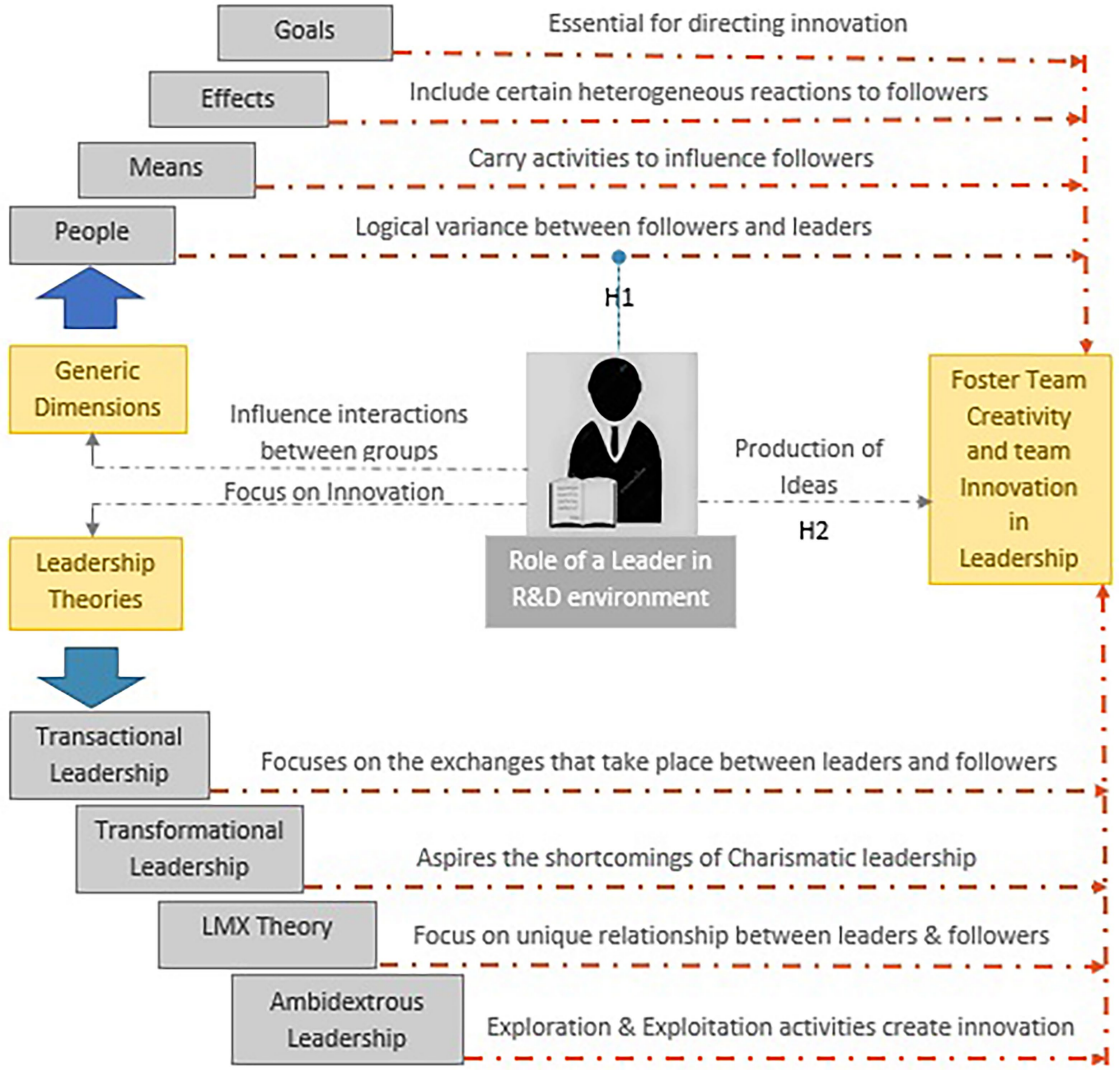
The role of a leader in R&D environments.

Although the concept of leadership is versatile, the person guiding the leadership role is named the “leader,” while the group guides are called the “followers,” although the same person can perform both functions simultaneously (Yukl, 2002; Gosling et al., 2009; Middlehurst et al., 2009; Griffith et al., 2018; Pelletier et al., 2019). Leaders can inspire followers to pursue collective values and aspirations as well as sacrifice egocentric needs and goals. These theories also reveal that leaders can invoke and regulate emotions – rather than rely on rational processes – to motivate other individuals (Moss and Ritossa, 2007, p. 433). In the light of Kesting et al. (2015), leadership means an expression of behavior that influences an individual’s attitudes and behaviors and interaction between groups (see Figure 1 below) for the motive of achieving goals. Therefore, there are four generic dimensions of leadership, i.e., people, means, effects, and goals (PMEG). People mean effects goals framework is not used by any researcher. Furthermore, Winston and Patterson (2006) also added that during the leading process, the leader allows the followers to be innovative and even self-directed within the scope of individual-follower assignments and allows the followers to learn from their own as well as others’ successes, mistakes, and failures along the process of completing the organization’s objectives.

The PMEG framework (Figure 1) highlights two central concepts considering the generic dimensions influencing business and the examined leadership theories in an R&D environment. Leadership means an expression of a particular behavior (Kesting et al., 2015) but considering this framework; we do not know how the PMEG framework will impact leadership in an R&D environment. However, a knowledge gap exists regarding how and to what extent the PMEG framework will impact leadership in an R&D environment (Fachrunnisa et al., 2019; Andrej et al., 2022). Correspondingly, Chemers (1997, p.1) defines leadership as “a process of social influence in which one person can enlist the aid and support of others in the accomplishment of a common task.” The occurrence of four generic dimensions in leadership (Kesting et al., 2015, p. 23) are implied as follows:

People – Leadership is a supra-individual concept that requires a logical distinction (which can be explicit or implicit, temporary or persistent) between leaders and followers, but without it, leadership is pointless. For instance, ethical leaders are likely to be people-oriented. Human rights, personality, capabilities, and learning, are likely to be promoted by ethical leaders who give their staff members a chance to learn the skills and information required for their jobs and put them in the appropriate positions. Additionally, they inspire followers to focus their skills in the appropriate direction for improved work performance. As a result, employees have the information, abilities, and motivation to act creatively and are then expected to do so in the workplace, fostering creativity (Kremer et al., 2019; Shafique et al., 2019).

Means – The nature of leadership is that leaders lead and carry out certain activities to direct or influence followers. The review will show that these means can include very heterogeneous activities like coaching, empowering, or even servicing, and there is no leadership without such activities. For example, the importance of employee effort in achieving company goals is highlighted by leaders with high ethical and moral standards; it has been observed. Employees are motivated by cognitive mechanisms to pay more attention to the worth of their job, which encourages them to generate and use original ideas to discover new ways to achieve organizational goals (Shafique et al., 2019; Khan et al., 2020; Lee et al., 2020).

Effects – The outcome of leading is to cause a reaction in the followers, i.e., to make them follow. The analysis will show that the effects can include heterogeneous reactions, like increased enthusiasm or commitment, the rational optimization of rewards, implicit convictions, etc. Without any result, leadership efforts go nowhere. It is noted in Shafique et al.’s (2019) study that employees are more likely to participate in knowledge development and dissemination in companies where ethical leaders create policies to facilitate knowledge sharing and foster a cooperative environment. The creation of such an atmosphere makes it easier for employees to connect and communicate, and it helps them build relationships of cooperation and trust with their colleagues and leader(s). Therefore, to sustain communication and promote cooperation, employees invest their time in knowledge creation, which increases the number of new ideas they bring to their workplace (Akbari et al., 2020; Khan et al., 2020).

Goals – Leadership is ultimately associated with specific goals. These goals can be broad visions of promising future states, but they can also be firm targets. In either case, leadership points towards a path. In the context of this paper and considering the above arguments, we claim our first hypothesis below, which is linked to RQ1:

*H1*: The PMEG framework will have a positive impact on leadership as leaders focus on those factors that influence an individual’s attitudes, behaviors, and interactions between groups.

Additionally, it builds followers’ performance, transforms personal values, and moves them toward high aspirations (Paulsen et al., 2013; Kremer et al., 2019; Shafique et al., 2019).

### Role and skills of the leader

One of the prominent roles leaders can play is to create an environment that will support the engagement of their subordinates and other people involved with tasks (effective teamwork; Amabile et al., 2004; Konradt, 2014; Mailhot et al., 2016). Leadership also includes encouraging followers and fostering auspicious conditions to carry out the work (Yukl, 2002; Bolden et al., 2009). Concepts of shared leadership (Konradt, 2014, p. 290) argue that leadership should not be conceptualized as a centralized downward process of influence on subordinates that is carried out by an appointed leader. Another prominent role of leaders is to create structures that will enhance creative activities to thrive and support creative endeavors (Anderson and West, 1998; Bain et al., 2001). More so, team leaders play a significant role in ensuring that projects, processes, and managing of resources are successful through efficient and flexible leadership (Magellan Horth and Vehar, 2015; Andrej et al., 2022). In that regard, it was revealed that a good leader not only challenges but also inspires work creativity (Sauermann and Cohen, 2010). Therefore, the technical skills of a leader not only impact the skillfulness of this individual, but it is an asset for a company towards promoting creativity and innovative performance of subordinates.

As a leader, having the necessary skill set to lead a supportive working environment are stepping-stones in initiating a creative environment for the subordinates. Yukl (2002) recognized that three primary skill sets are essential for leaders. First, technical skills include knowledge about methods, processes, and equipment for carrying out particular activities in the given unit. Second, cognitive or conceptual skills comprise the general analytical ability, logical thinking, proficiency in concept formation, and conceptualization of complex and ambiguous relationships. They include, for example, good judgment, intuition, or creativity. Finally, interpersonal skills like empathy, tact, and diplomacy are essential to follow human behavior and interpersonal processes and thus influence others (Yukl, 2002; Khan et al., 2020). Hackman and Hackman (2002) also proposed that both displays of “diagnostic skills” and “execution skills” from the leader are decisive. While “diagnostic skills” helps leaders to distinguish important events happening to the team/organization from noises, “execution skills” are needed for effective team leadership.

### Leadership in the research and development (R&D) environment

R&D organizations and groups are dynamically involved in developing innovative products, services, technologies, and processes that may foster superior performance at lower costs. Moreover, focusing on the above leads to coherent ideas and methods that foster technological information in the R&D environment (Gupta and Singh, 2015; Du et al., 2020; Rêgo et al., 2022). Therefore, the production of ideas (Figure 1) for organizational products and services is one of the critical growth production drivers, i.e., employee creativity. It has been stated that creativity is a vital component of innovation. The central idea of social cognitive theory is self-efficacy. Bandura (1997) emphasized that strong self-efficacy in individuals is a prerequisite for creativity and the discovery of new knowledge. The most important component affecting conduct is self-efficacy. A particular form of self-efficacy called creative self-efficacy pertains to people’s beliefs that they can engage in creative action (Cai et al., 2019).

Additionally, Zheng et al. (2010) define R&D teams as groups focused on creating original innovations and expanding existing ones in their field of interest. These teams are composed of highly educated and creative members (Berson and Linton, 2005; Bagheri and Harrison, 2020). However, Gupta and Singh (2015) further proclaim that the key factors that sustain R&D professionals in creative behavior imply enhancing organizational competitiveness. Due to the unique and uncertain process of innovation, R&D must manage different work units where the work is routine. Furthermore, R&D leaders are more professional than organizational professionals and usually select technical expertise for leadership skills. In Gumusluoglu et al.’s (2017) view, R&D leaders should facilitate innovation behaviors through different recognitions, such as team innovative behavior (generation and implementation of new ideas) through team identification and cross-team innovative action (exchange of resources, coordination with other teams to facilitate and implement innovations) through building department identification (Gilbert and Basran, 2019; Du et al., 2020).

Empowering leadership (EL), examined by Amundsen and Martinsen (2015), mentions EL as behaviors that share power with subordinates and lead others. This approach promotes self-leadership (the process of controlling their behavior and influence, leading themselves through a specific set of cognitive and behavioral strategies) among employees. By and large, R&D environments offer a unique challenge to leadership (Bryman and Lilley, 2009; Basran et al., 2019; Du et al., 2020; Ackaradejruangsri et al., 2022). In addition to building up a favorable climate for creativity, leaders’ roles are also to ensure their subordinates remain actively involved in their work and try to generate innovative products, techniques, and methods as means to remain competitive (Shalley and Gilson, 2004; Xenikou, 2017; Wang et al., 2019). According to Gupta and Singh (2015), an R&D leader’s behavior makes things work and avoids wasting time, labor, and capital. These R&D professionals constitute unique leadership challenges essential to foster team creativity. Most studies testing leadership’s impact on employee creativity have found the two-factor behavioral conceptualizations of leadership (e.g., initiating structure and task-oriented). However, only a limited number of studies have been conducted focusing on R&D organizations and contexts compared to the flood of studies on leadership in general (Elkins and Keller, 2003; Xenikou, 2017; Tong, 2020).

Interestingly, teams in the R&D environment are typically cross-functional, bringing together a combination of scientists, technicians, engineers, and specialists (Denison et al., 1996). Also, Lisak et al. (2016) support that R&D leaders foster the team’s innovation goals and motivate a team to adapt to understanding within teams, which will positively affect an organization. In this regard, the managerial practices that have been recognized to foster this type of climate include autonomy of subordinates, personal recognition, development of group cohesion, and resource maintenance.

### Different types of leadership applied in R&D

Over the past 30 years, research has shown the crucial role leaders play not only in their subordinates’ motivation and efficacy but also in their creativity and innovation performances. For example, Tierney et al. (1999) demonstrated that creativity and inventions increased when leaders and followers pursued productive exchange relationships. Interestingly, Michalko (2001) showed that creativity was more likely to occur when leaders avoid letting their judgment emerge when a follower speaks. Various leadership theories have been described, including authoritative, coaching, and democratic (Goleman, 2000; Al Harbi et al., 2019; Perpék et al., 2021). However, only a few have tested in R&D environments. Additionally, Yunlu and Murphy (2012) explore that during the recession, R&D generally decreases, but the researchers find support for R&D growth, which positively correlates with overall economic growth.

Early work from Pelz (1963) on scientists from 20 research laboratories based in the United States has shown that interactions with a given group’s leader can positively influence creativity, and this was particularly prominent in junior scientists. Further work also emphasized that the supervisory style employed by the leaders toward creativity was positively related to performance (Mednick and Mednick, 1967; Xenikou, 2017; Tong, 2020). Research has also shown that leaders play a significant role in exploiting the outcomes of R&D projects by triggering cooperation among teammates and communicating and sharing the body of knowledge and skills (Gillespie and Mann, 2004; Carayannis et al., 2021; Perpék et al., 2021) efficiently. It has been proposed that the most influential leadership theories are the transformational and the transactional leadership theories (Bass, 1985), which are related to innovation performance both at the individual and the team levels and when whole organizations are the unit of measurement.

The concept of transactional leadership was first introduced and discussed with transformational leadership by MacGregor Bums (1980). There have been discussions about the two leadership styles, but as Kesting et al. (2015) clarify, transactional leadership does not focus on change as transformational leadership does (Moss and Ritossa, 2007; Lin et al., 2020; Ali et al., 2021). Particularly transactional leadership focuses on the exchanges between followers and leaders, as shown in Figure 1 above (McCleskey, 2014). Additionally, transformational leadership about innovation and change is the most researched leadership style (Bolden et al., 2009; Kesting et al., 2015; Oeij et al., 2017; Alghamdi, 2018). Paulsen et al. (2013) claim that transformational leadership focuses on the specific role of a leader in promoting organizational and personal change to assist employees in exceptional performance. Also, it empowers the team members and moves them by providing them with idealized charisma and inspirational motivation. Additionally, it builds followers’ performance, transforms personal values, and moves them toward high aspirations. However, there is no description of how can/whether transformational leadership can aspire to the shortcomings of charismatic leadership (Figure 1). Similarly, in a prolonged study, Chaubey et al. (2019) found that transformative leadership encourages staff innovation. To lead for creativity, one must inspire followers to come up with creative ideas. As a result, leadership is a crucial requirement for creative outcomes. The creativity of organizational employees is positively impacted by transformational leadership (Al Harbi et al., 2019).

Leadership can enhance a follower’s sustainability, facilitating an employee’s performance and creativity; for this, the leader-member exchange (LMX) theory was introduced (Smothers et al., 2012; Oeij et al., 2017; Alghamdi, 2018). It focuses on the unique work relationship between leaders and their subordinates (as shown in Figure 1) rather than on variables such as traits or behaviors. LMX theory is the quality of exchanges that develop between leaders and followers to conduct creativity (Smothers et al., 2012; Qu et al., 2017; Martin et al., 2018; Soleas, 2020). Another essential theory was introduced in management, which relates to the ability to excel at explorative and exploitative organizational strategies known as Amberdexidery (Raisch and Birkinshaw, 2008; Xenikou, 2017; Tong, 2020). Keller and Weibler (2015) support that exploration (comprises activities) and exploitation (captures activities) are the two organizational patterns of ambidextrous pursuit that are essential for the superior performance of a firm. Further, Zacher and Rosing (2015) have used the term primarily to mention exploration and exploitation activities (Figure 1). Concerning such activities, Sinha (2016) confirm Zacher and Rosing’s argument by supporting that the ability of these existing resources and activities create platforms for future growth through experimentation and innovation, which further focus on organizational performance in both the long and short-term.

### Factors influencing creativity and innovation

Research and development depend significantly on creative minds that will be able to fashion tomorrow’s innovations. Gumusluoglu et al. (2017) examine that if organizations want to foster creativity and innovative behavior, it is particularly essential to enhance leadership through the coordination of their collective actions as well as expertise. Leadership that stimulates innovation has been a subject of research, and the mechanisms for its connection with the innovation process include creativity and implementation of creative ideas (Černe et al., 2013, p. 64; Ackaradejruangsri et al., 2022). Creativity is essential in the production of ideas by employees, as well as critical to the leader trying to enhance work settings. Among several factors, the output of creativity is highly influential in the supportive behaviors of leaders (Li et al., 2018; Bagheri and Harrison, 2020; Soleas, 2020).

**Figure 2:**
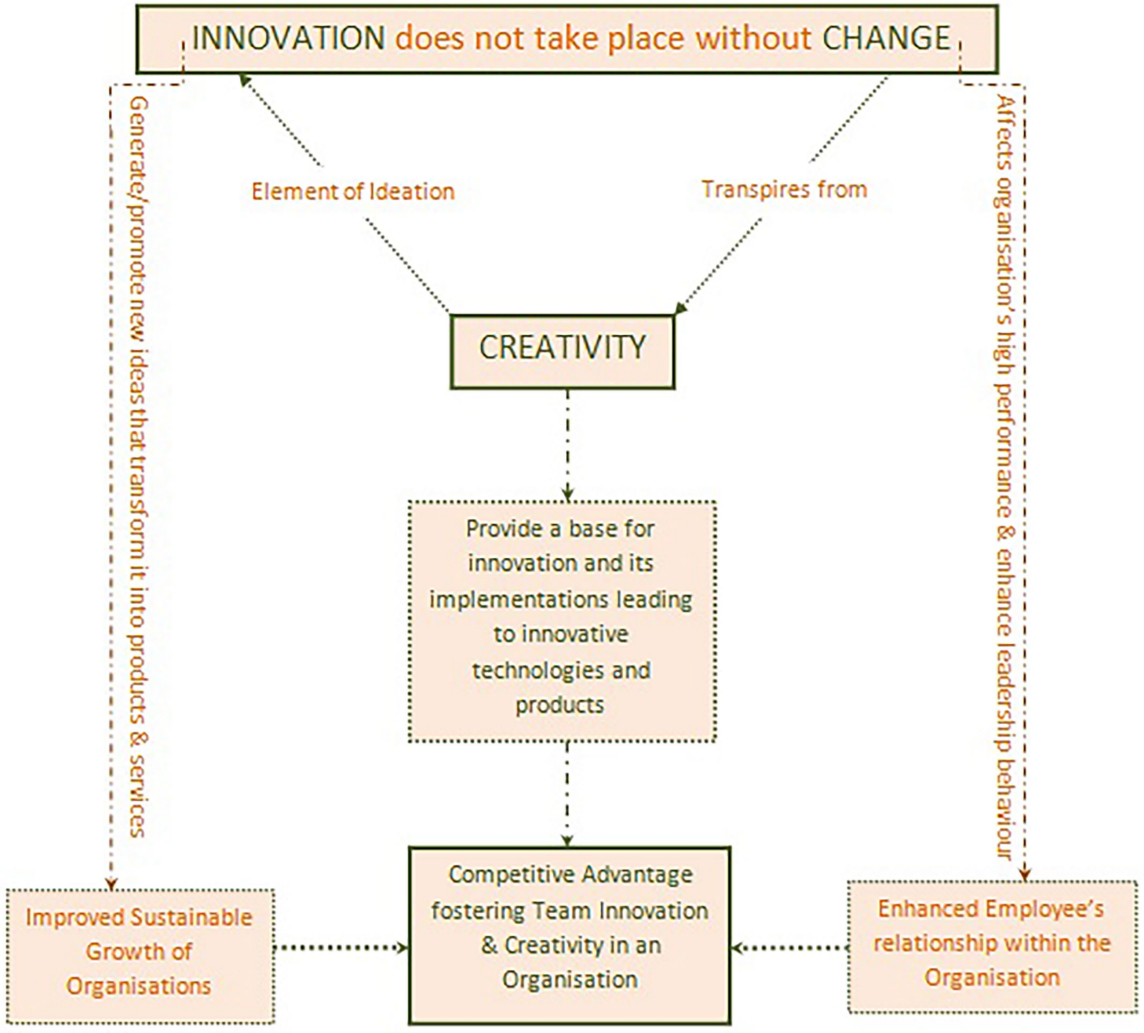
Dynamics between creativity, innovation and change in R&D settings.

Additionally, Rosso (2014) states that creativity is defined as the solutions to produce valuable ideas in an organizational setting as a critical implementation of ideas. Hence, creativity is an analytical source for competitive advantage and innovative products and technologies. Furthermore, Ghosh (2015) supports that creativity is a composite but still constructed in several ways. Creativity is an ingredient for innovation (see Figure 2) that implies the successful implementation of ideas. Hon and Lui (2016) confirm Ghosh’s argument by supporting that creativity leads individuals to contribute their skills, ability, and willingness to work. Thus, Ghosh (2015) claims that creative ideas provide a base for innovation and its implementations.

Correspondingly, Kesting et al. (2015) state innovation as a multistage process that transforms ideas into products and services that further successfully compete in the market. Therefore, creativity is necessary but not essential for some conditions. Thus, change is needed, and innovation does not occur without change (see Figure 2; Jiang et al., 2017; Ali et al., 2021). Moreover, it is presumed that innovation consists of a variety of activities. One approach to structure this distinction’s complexity is the various innovation phases, such as implementation and ideation or the difference between commercialization and development. Creativity is just an element of the ideation or development stage related to innovation. Conversely, Lisak et al. (2016) support that the information perspective on team creativity is examined by leaders’ positive effects on team innovation.

### Personal traits affecting creativity and innovation

Both creativity and innovation have now become critical positive factors of organizational performance (Arad et al., 1997; Drazin et al., 1999; McMahon and Ford, 2013; Yi et al., 2017). Several core personality traits such as broad interests, independence of judgment or a strong sense, skills like problem-solving or engaging in divergent thinking (Amabile, 1988; Mumford et al., 1997; Vincent et al., 2002), as well as experience in a field (Weisberg, 1999), influence creativity at the individual level, have been identified as potentially triggering creativity and innovation. The interview-based study by Amabile (1988) showed that some qualities that promote creativity, including persistence, curiosity or intellectual honesty, strong self-motivation, unique talents, attraction to challenges, and expertise in a particular field, positively encourage creativity. Additionally, the same study showed that specific qualities of the environment play a role in creativity. For example, the factors detected as the most important by the scientists interviewed were decision freedom, project management, sufficient resources, and encouragement, with 74, 65, 52, and 47%, respectively. Further, general organizational performance has also been associated with creativity and innovation in corporations (Irwin et al., 1998; Capron, 1999; Bagheri and Harrison, 2020).

### Creativity and innovation in the R&D environment

Creative thinking and development of innovations such as new methods of analysis are of utmost importance to develop the liability of the service research laboratory and thus build up the client base of the laboratory. Also, the development of new methods offers the potential for scientists to continue publishing in high-standard peer-reviewed journals and give them chances to progress in their career path. Although publishing in scientific journals might not be the most important motive of scientists working in service-based laboratories, this still provides a stable motivational ground. Recent work by Roach and Sauermann (2010) revealed that incentives influence the decision to work in academia or the industry. The authors collected 472 responses to their questionnaire from Ph.D. students based in North Carolina (US). They showed that scientists motivated by the freedom to choose their research projects, the ability to publish, and the aspiration to perform necessary research tend to pursue careers in academia. At the same time, the industry attracts those motivated with a competitive salary, access to equipment, and the possibility to carry out applied research. In the light of Shalley and Gilson (2004), innovations also offer the possibility for service-based laboratories to remain competitive; for this, managers or leaders need to ensure their employees are actively involved in their work.

The ability to innovate is hampered by external and internal organizational constraints, which are significant organizational factors in long-term competitive positions. An organization can achieve long-term success if and only if it consistently maintains new products and services to satisfy the needs of its clients. A company must acknowledge the importance of creativity and innovation, both of which are crucial elements of the same process, in order to be successful. Having ideas does not mean they have to be put into action; rather, an individual’s excitement for networking and doing so leads to execution. Individuals or groups can produce creativity, independent of their functional specialties or hierarchical levels within the organization (Hughes et al., 2018; Chaubey et al., 2019; Shafi et al., 2020).

Creativity and innovation capacity are necessary/essential elements in a knowledge-based economy. Furthermore, it is a primary competitive advantage source as well as a key to high productivity (Figure 2). Likewise, Wang et al. (2015) claim that the organizational climate has proven to be the best factor that exploits employees’ innovation. Comparatively, Hon and Lui (2016) support that organizational leadership has devoted attention to innovation models and creativity. Further, Wang et al. (2015) support Hon’s argument by confirming that innovative behavior refers to the promotion, generation, and realization of the latest and new ideas within a workgroup or organization. Therefore, the individual’s innovation success depends on an employee’s relationship with the organization that provides information, resources, support, and inspiration that helps innovators to promote and develop new ideas.

### Influence of leadership on creativity and innovation

Innovative achievements can only happen when team members bring about ideas and efforts. Also, Vogel and Fischler-Strasak (2014) stated that innovation is essential for the sustainable growth of large enterprises as they are misdesigned for innovation. However, in most organizations’ cultures, innovation is not impossible but challenging. Thus, to stay competitive, every organization should nurture innovation appropriately and particularly (see Figure 2). Likewise, Dunne et al. (2016) claim that innovation is particularly essential for small firms resulting in the efficient production of their products and services. Further, some precursor of innovation includes information, marketing capabilities, and communication technologies.

As organizations encounter rapid changes in technology and economic forces, employee creativity is regarded as the potential resource for an organization’s survival (generation of ideas for products, services, and practices in the workplace). Among these factors, leadership plays a vital role in employee creativity to facilitate an organization’s goals (Yi et al., 2017). Organizational factors also restrain the creativity of individuals working together. Moreover, organizational creativity has investigated various factors taking into consideration the employee surrounding, employee rewards, job characteristics, and organizational goal setting, which leads to creative outcomes (McMahon and Ford, 2013; Felix et al., 2019). According to Noor (2013), the relationship between leadership practices and innovative work behavior is shown. The purpose of organizational innovation is affected by individual attributes like resistance to change and leadership.

Additionally, leadership can influence innovative behavior; this is agreed upon by most researchers. However, giving a reward to the employees was related to innovativeness, but this does not correlate with innovativeness. Correspondingly, stimulating new ideas and solutions by enhancing ideas and goals for work are the two essential processes for leadership and innovation. The importance of leadership and integration for innovation is comprehensive and targeted, which attain team innovation to promote creativity (Noor, 2013; Hughes et al., 2018; Jiang and Chen, 2018). Furthermore, Yuan and Woodman (2010) have also found that innovation might be enhanced when leaders expect high performance and then recognize the work done. It is the role of managers to create an environment that fosters creativity. Additionally, Jaiswal and Dhar (2016) state that leaders play an essential role in determining employees’ creativity. For instance, a leader’s behavior is critical because it determines the creativity among individuals in the work environment. Therefore, employee creativity is fostered through a distinct leadership style, i.e., transformational leadership.

Despite no difference, creativity and innovation are studied separately with little or no amalgamation. There is a default as team innovation, and creative endeavors are essential for an organization to thrive. But despite the role of leaders, those energetic team innovation and creativity, no research simultaneously influences both outcomes. Now the motive is to foster both team innovation and employee creativity simultaneously. For this, the role of servant leadership on employee creativity is examined. Therefore, a holistic approach, i.e., servant leadership, encompasses a leader’s emotional, rational, and moral dimensions that further enhance followers’ growth and capabilities. Servant leadership shares some similarities with transformational leadership, but these behaviors are motivated more by organizational goals than followers’ performance and development, as a transformational leader does (Yoshida et al., 2014; Lin et al., 2020). It has thus suggested that leadership styles that offer team members an active role in the leader-follower work relationship would be more suited to R&D settings.

The conceptual framework (Figure 2 below) highlights another concept, exploring one more research gap, i.e., how and to what extent the production of ideas in an organization leads to growth, fostering team innovation and creativity. As mentioned in the literature above, organizations encounter rapid changes in technology and the economy; employee creativity is regarded as the potential resource for an organization’s survival (generation of ideas for products, services, and practices in a workplace). Among these factors, leadership plays a vital role in employee creativity to facilitate an organization’s goals (Yi et al., 2017; Amah and Oyetuunde, 2020). Correspondingly, stimulating new ideas and solutions by enhancing ideas and goals for work are the two essential processes for leadership and innovation. The importance of leadership and integration for innovation is comprehensive and targeted, which attain team innovation to promote creativity (Noor, 2013; Jiang and Chen, 2018). Further, these arguments lead to the second hypothesis below, which is linked to RQ2:

*H2*: Production of ideas will impact positively on leadership, leading to growth and competitive advantage of the organization.

Thus, the outcome of the conceptual framework developed is improved sustainable growth and enhanced employee relationship within the organization, further leading to the organization’s competitive advantage.

## Research methodology

### Overview of the research approach

A quantitative analysis was plausible over qualitative research mainly because the survey was conducted using a single technique, employing a questionnaire (Bryman, 2006). Quantitative researchers attempt to study the phenomena of their interest, i.e., standardized surveys and other quantitative measuring devices are often used to carefully measure what is observed between different groups, companies, countries, or organizations, which is best understood through the survey approach (Amah and Oyetuunde, 2020). Further, the cross-sectional survey helps the researchers analyze the relationship between the variables described in the previous sections. Quantitative research is appropriate for this study because questionnaire items are selected after checking the principal component analysis (PCA) and confirmatory factor analysis (CFA; Chevallier et al., 2016). Further, Eikebrokk and Olsen (2005) support that quantitative research methods are essential for hypotheses testing as the dependent variable is measured with reflective indicators of each four dimensions of success, such as efficiency, novelty, lock-in, and complementarities. Each dimension’s success is necessary because it is constructed as the average item score. Moreover, Rahman (2016) supports that this method uses statistics data analysis, helping researchers understand several principles and elements of the data while utilizing measurable data to originate facts and uncover patterns in research.

Demographics and professional data were assessed in terms of ratios of total participants and included analysis of participants based on gender, age, nationalities, and continent representation. Further, gender representation was assessed by participants based on educational level, professional experience, professional role, and professional entities. Data distribution was statistically evaluated using standard statistical parameters such as calculating data mean, standard deviation, minimum, median, and maximum values. The six-point Likert scale was grouped into three categories, “the negative feeling,” which included the “not at all” (represented by 1) and “to almost no extent” (represented by 2) responses, “the neutral feeling,” composed of the answers “to a slight extent” (represented by 3) and “to a moderate extent” (represented by 4) and “the positive feeling” category, that correlated to “a great extent” (represented by 5) and “to a very great extent” (represented by 6) responses. These three groups allowed for a direct comparison against qualitative data analysis (Amabile et al., 2004). Finally, correlations among the different variables (questions/statements in the survey) were calculated to assess relatedness between statements. The correlation coefficient measures the linear relationship between two variables, always between −1 and +1. A correlation of +1 denotes that the two variables under consideration are perfectly related in a linear mode, while a −1 means the variables are perfectly related in a linear negative sense, and 0 denotes no linear relatedness between variables (Felix et al., 2019). All calculations were carried out using Excel (Microsoft Office).

### Sampling

A representative subset of the participants is chosen to complete the cross-sectional survey. The subset includes a vast majority of the participants (86.2%) who worked in an academic environment, only 10.8% worked in the industry, and 3% were employed in independent entities. About 20% of participants were between 21 and 30 years old, of which 69% were females, 19% percent were aged between 41 and 50 years, and about 67% were males. Demographically, 24 nationalities were represented in the survey, with Zimbabwean (19.7%), German (12.1%), British (10.6%), and French (9.1%) nationalities being the most represented. Noteworthy, six continents, namely Africa, Australia, Asia, Europe, America, and North America, were represented, giving a global perspective and a global scale of the way people from different nationalities and different continents perceive leadership and its role in creativity and innovation.

Regarding the education level, most participants (75%) obtained a Ph.D. as their highest qualification, and 20% had a Master’s degree. The remaining participants (about 5%) had an Undergraduate degree. Most participants had 1–5 years (37.5%) of professional experience, followed by 11–15 years (26.6%). Only 3% were juniors and had less than 1 year in professional environments. Further, when connecting the professional experience to the professional role, the results indicate that many participants were experienced staff (35.8%).

Demographically, participants from the academic research environment were randomly recruited to participate in this study, which was designed to gather views on the role of leadership, including team leadership, to promote creativity and innovation in academic scientific environments. The survey was developed with a total of 64 participants affiliated with the scientific community completing the survey. The large proportion of females in the student category correlates with the observation that most women in the study reached 1–5 years of experience. The results presented here suggest that professionals in academia must obtain PhDs to enter middle-range or high-range managerial positions. This agrees with the current data where more males holding PhDs had senior administrative positions than females.

### Data collection method

The survey was powered using Google forms, an online application, for simple accessibility, ensuring confidentiality and anonymity, and obtaining a high enough response rate for quantitative data analysis. This survey formed the basis of the quantitative-based data analysis allowing inferring statements. The number of participants needed to be large enough to ensure the accuracy of conclusions and limit bias (Easterby-Smith et al., 2012). This survey intended to “generalize from a sample to a population” (Babbie, 1990). Participants in the study were personally invited in participate in the survey *via* email. The invitation included an explanation of the study, and that confidentiality was guaranteed, the aim of the study as well as the link to the survey. Participation in the survey was completely voluntary and anonymous. Hence, it is not all invited participants responded to the survey. Only filled questionnaires were taken into consideration. Data from every question and every participant was converted to a table in Excel (Microsoft Office) using the “View Responses” tool on Google Forms and limited errors in data file transcription.

## Analysis of findings

### Personal work perception of individual participants

In the following section of the survey, participants were invited to give their sentiments about eight statements describing their perceptions at work (Table 1). Here, participants had to choose from a six-point Likert scale to answer qualitatively about their feelings. For this set of questions, the maximum measure selected was “to a very great extent” designated by value 6, while the minimum ranges from “not at all” (1) to “to a slight extent” (3). Table 1 shows that respondents rated “individual characteristics necessary for creativity and innovation” as the most important factor (mean: 5), followed by “position in which creativity and innovation thinking are necessary” (mean: 4.83) and “you like your working environment” (mean: 4.78). On the other hand, the least two important factors where “you find your work rewarding” (mean: 4.25) and “you feel that you have support from your team and colleagues” (mean: 4.33).

**Table 1:** Summary of statistics indicating the mean, standard deviation, minimum, median, and maximum values of the participants’ responses.

Variable	Statement	Mean	Standard deviation	Min	Median	Max
**Yourself and your work**						
You feel motivated to go to work	8	4.58	0.87	2	5	6
You like your working environment	9	4.78	0.95	2	5	6
You find your work rewarding	10	4.25	1.11	1	4	6
You find your work challenging	11	4.56	1.11	1	5	6
You are employed in a position in which creativity and innovative thinking are necessary	12	4.83	1.11	2	5	6
You feel that you have the tools necessary to perform well at work	13	4.59	1.08	1	5	6
You feel that you have support from your team or colleagues	14	4.33	1.16	2	4	6
You feel you have the individual characteristics necessary for creativity and innovation	15	5.00	0.84	3	5	6
**About your manager’s style**						
Planning and organization are clear and fair to all group members	16	4.19	1.28	1	4,5	6
Roles and objectives are clearly planned and set	17	4.28	1.12	1	4	6
Communication and interaction are done to all group members through different means	18	4.63	1.00	2	5	6
Group conflicts are solved proactively by identifying the cause and finding solutions	19	4.02	1.18	1	4	6
The work progress of individuals and teamwork are monitored closely	20	4.09	1.22	1	4	6
All team members are motivated and inspired	21	3.97	1.21	1	4	6
Subordinates are consulted and used to brainstorm before implementing changes that affect the group	22	3.81	1.39	1	4	6
Substantial delegation is being offered to provide extra responsibilities and empowering	23	3.70	1.34	1	4	6
Regular constructive feedback and support are being offered	24	4.05	1.52	1	4	6
Coaching and mentoring are provided for personal development and career progression	25	3.92	1.46	1	4	6
Conflicts are well managed	26	3.83	1.32	1	4	6
Team cohesion and spirit are built and maintained	27	3.84	1.43	1	4	6
A network is developed, and communication is maintained through direct and indirect interactions	28	4.05	1.16	1	4	6
Appreciation of individual performance and contributions are expressed	29	3.84	1.34	1	4	6
Rewards like a salary increase or career advancement are offered	30	2.78	1.39	1	3	6
**About the support to nurture creativity and innovative spirit provided to yourself and the team**						
The working environment created is propitious to creativity and innovation	31	4.13	1.35	1	4,5	6
The level of autonomy offered is critical for creativity and innovation	32	4.56	1.04	2	5	6
The possibility to free some time from tasks to reflect and think helps your creative spirit	33	4.39	1.15	1	5	6
The manager offers me enough flexibility necessary to develop creative thinking	34	4.50	1.15	2	5	6
The manager helps the team to remain up to date with technology and know-how in the field and related competitive disciplines	35	4.06	1.44	1	5	6
The manager embraces multicultural community to strengthen the team’s capabilities	36	4.30	1.51	1	5	6
The manager’s style and interpersonal behavior directly help the team to adapt to change and integrate new ideas	37	3.88	1.32	1	4	6
Openness and opportunities are nurtured within the team to demonstrate freely curiosity and introduce new ideas	38	4.00	1.38	1	4	6
High ethical standards and social responsibility are supported to maintain confidentiality	39	4.30	1.42	1	5	6

Besides, the six measures were grouped into three categories. The first category corresponds to negative feelings and comprises “not at all” (1) and “to almost no extent” (2) responses. The second group depicts neutral sentiments and contains the answers “to a slight extent” (3) and “to a moderate extent” (4). At the same time, the third section integrates positive feelings with “to a great extent” (5) and “to a very great extent” (6). Responses showed that most respondents were neutral or positive about their work. 58% of the participants were motivated to go to work (s8), and about 67% liked their working environment (s9). A large proportion found their job rewarding (s10), but the ratio of neutral feelings was greater (48.4%) than the positive responses (43.8%). 56.3% of the participants found their work challenging (s11) and received relative support from their colleagues (s14; 43.8% positive and 50% neutral). Interestingly, 71.9% responded positively about the necessity for creativity in their current positions (s12), and 78.1% feel they have the characteristics for creativity and innovation (s15). None of the respondents felt pessimistic about their capacity for creativity and innovation. Of all participants, 68.8% thought they had the necessary tools to perform well at work.

The analysis of the participants’ work perception revealed that although they felt they had the personal capacity and motivation for creativity and innovation and the tools to succeed, there was generally low support from colleagues. This can be caused by high competitiveness within the academic sector and a limited drive for collaboration (Carson et al., 2013). This aspect has, however, been shown to be critical to encouraging creativity in previous studies (Payne, 1990; Amabile et al., 1996). This suggests that the lack of formal teamwork may hinder exposure to novel ideas, reduce commitment to projects and, in turn, reduce individuals’ motivation over time. It may also minimize creative thinking (Abra, 1994; Cagliano et al., 2000) suggested that this requires acquiring and utilizing expertise and collaborative efforts. Thus, academic leaders must focus on developing teamwork, collaboration, and team spirit to benefit creativity and innovation in their teams.

### Effect of a leader’s behavior and management style on subordinates

On assessing the manager’s leadership style, 15 statements were asked for an assessment, similar to the above section. Data analysis revealed that the minimum mean was given to the statement about “opportunities for rewards,” with a mean value of 2.78. On the other hand, participants rated the statement on “communication and interaction” the highest, with a mean value of 4.63 (Table 1).

Statements in this section were derived from the Managerial Practices Survey (MPS) and the categories defined by Yukl (2002), except “Managing conflicts” and “Team-building” which were split into two separate categories. In the present study, the four categories that raised the most positive feelings were “Informing” (64.1%), “Planning and organizing” (50%), “Supporting” (46.9%), and “Clarifying roles and objectives” (45.3%; Table 2). In the study by Amabile et al. (2004), the categories of “Monitoring,” “Consulting,” “Supporting,” and “Recognizing” were found to be positively correlated to leader support. Of these, only the category of “Supporting” was associated with a positive feeling in the present study. The other three categories, namely “Monitoring,” “Consulting,” and “Recognizing,” were found to bring neutral feelings at 50, 40.6, and 43.8%, respectively, as the most common consensus among the participants.

**Table 2:** Categories of an individual’s feelings and perspectives about work.

Statements	MPS category	Negative^1^	Neutral^2^	Positive^3^
About yourself and your work				
You feel motivated to go to work	n/a	2 (3.1%)	25 (39.1%)	37 (57.8%)
You like your working environment	n/a	2 (3.1%)	19 (29.7%)	43 (67.2%)
You find your work rewarding	n/a	5 (7.8%)	31 (48.4%)	28 (43.8%)
You find your work challenging	n/a	3 (4.7%)	25 (39.1%)	36 (56.3%)
You are employed in a position in which creativity and innovative thinking are necessary	n/a	3 (4.7%)	15 (23.4%)	46 (71.9%)
You feel that you have the tools necessary to perform well at work	n/a	5 (7.8%)	15 (23.4%)	44 (68.8%)
You feel that you have support from your team and/or colleagues	n/a	4 (6.3%)	32 (50.0%)	28 (43.8%)
You feel you have the individual characteristics necessary for creativity and innovation	n/a	0 (0.0%)	14 (21.9%)	50 (78.1%)
About your manager’s style				
Planning and organization are clear and fair to all group members	Planning & organizing	7 (10.9%)	25 (39.1%)	32 (50.0%)
Roles and objectives are clearly planned and set	Clarifying roles & objectives	6 (9.4%)	29 (45.3%)	29 (45.3%)
Communication and interaction are done with all group members through different means	Informing	1 (1.6%)	22 (34.4%)	41 (64.1%)
Group conflicts are solved proactively by identifying the cause and finding solutions	Problem-solving	8 (12.5%)	32 (50.0%)	24 (37.5%)
The work progress of individuals and teamwork are monitored closely	Monitoring	6 (9.4%)	32 (50.0%)	26 (40.6%)
All team members are motivated and inspired	Motivating & inspiring	7 (10.9%)	33 (51.6%)	24 (37.5%)
Subordinates are consulted and used to brainstorm before implementing changes that affect the group	Consulting	14 (21.9%)	26 (40.6%)	24 (37.5%)
Substantial delegation is being offered to provide extra responsibilities and empowering	Delegating	14 (21.9%)	28 (43.8%)	22 (34.4%)
Regular constructive feedback and support are being offered	Supporting	10 (15.6%)	24 (37.5%)	30 (46.9%)
Coaching and mentoring are provided for personal development and career progression	Developing & mentoring	9 (14.1%)	29 (45.3%)	26 (40.6%)
Conflicts are well managed	Managing conflicts	10 (15.6%)	30 (46.9%)	24 (37.5%)
Team cohesion and spirit are built and maintained	Team building	12 (18.8%)	30 (46.9%)	22 (34.4%)
A network is developed, and communication is maintained through direct and indirect interactions	Networking	7 (10.9%)	34 (53.1%)	23 (35.9%)
Appreciation of individual performance and contributions are expressed	Recognizing	10 (15.6%)	28 (43.8%)	26 (40.6%)
Rewards like a salary increase or career advancement are offered	Rewarding	28 (43.8%)	29 (45.3%)	7 (10.9%)
About the support to nurture creativity and innovative spirit provided to yourself and the team				
The working environment created is propitious to creativity and innovation	n/a	10 (15.6%)	22 (34.4%)	32 (50.0%)
The level of autonomy offered is critical for creativity and innovation	n/a	4 (6.3%)	18 (28.1%)	42 (65.6%)
The possibility to free some time from tasks to reflect and think helps your creative spirit	n/a	7 (10.9%)	21 (32.8%)	36 (56.3%)
The manager offers me enough flexibility necessary to develop creative thinking	n/a	6 (9.4%)	16 (25.0%)	42 (65.6%)
The manager helps the team to remain up to date with technology and know-how in the field and related competitive disciplines	n/a	10 (15.6%)	21 (32.8%)	33 (51.6%)
The manager embraces multicultural community to strengthen the team’s capabilities	n/a	10 (15.6%)	18 (28.1%)	36 (56.3%)
The manager’s style and interpersonal behavior directly help the team to adapt to change and integrate new ideas and information	n/a	12 (18.8%)	25 (39.1%)	27 (42.2%)
Openness and opportunities are nurtured within the team to demonstrate freely curiosity and introduce new ideas	n/a	13 (20.3%)	22 (34.4%)	29 (45.3%)
High ethical standards and social responsibility are supported to maintain confidentiality	n/a	9 (14.1%)	20 (31.6%)	35 (54.7%)

^1^Negative feeling combines answers ‘not at all’ and ‘to almost no extent’; ^2^Neutral feeling combines answers ‘to a slight extent’ and ‘to a moderate extent’; ^3^Positive feeling combines answers ‘to a great extent’ and ‘to a very great extent’.

On the other hand, Amabile et al.’s (2004) study revealed that the category of “Roles and objectives,” “Problem solving,” and “Monitoring” were negative forms of support. However, in the present study, none of these categories raised high negative ratios. The negative impact for “Roles and objectives” was observed in 9.4%, “Problem solving” in 12.5%, and “Monitoring” in 9.4% of the participants. Solving problems is critical in R&D environments and scoring a low negative ratio signals that leadership in this sector does confront complex, ill-defined problems, although, in this study, the category does not seem to critically depend on support. Team leadership has been posited as critical to diagnose and solve problems that keep the team from realizing their full potential (Zaccaro et al., 2001). It has been pointed out that influential team leaders must know how to solve problems accurately, intervene effectively (Shea and Guzzo, 1987), and use the team’s combined expertise to analyze problems to design effective solutions (Hiller et al., 2006). The three categories that showed the most important negative feelings were “Consulting,” “Delegating,” and “Rewarding” with 21.9, 21.9, and 43.8% of the total participants.

Interestingly, the category “Consultation” was detected as one of the positive forms of behavior. The discrepancies between the study of Amabile et al. (2004) and the present study are probably due to the different setups of the two studies and the focus on R&D-based participants in the present study. In the study, data were collected through an online survey, while the study by Amabile et al. (2004) was derived from a quantitative analysis of daily diaries. Additionally, the pool of participants was a much wider participant group in the study of Amabile et al. (2004; i.e., 238 employees) against 64 in the present study. However, the results suggest that academic environments might have very particular managerial practices.

The observation of the categories of “Informing,” “Planning and organizing,” “Supporting,” and “Clarifying roles and objectives” as the four most positive managerial practices suggest that managers in R&D environments can organize and communicate to their subordinates effectively in addition to providing leadership support and planning. These categories show that the leaders provide resources and take action to secure them using planning, organizing, and supporting subordinates (Shea and Guzzo, 1987). As the authors argued, providing adequate resources is beneficial as they facilitate the completion of tasks and show support to the team. It has also been proposed that providing resources can motivate teams because it communicates that the work is valued and appreciated and facilitates work efficiency (Morgeson et al., 2010).

However, the most negative categories, “Consultation” and “Delegation,” suggest a risk of reduced team involvement in important tasks as subordinates are given low opportunities to evolve and limited empowering prospects. This may impact motivation in the long run, and thus overtime workers will become less actively involved and motivated in their work, although Shalley and Gilson (2004) stated that this was an essential role of leaders. Finally, the fact that “Rewards” was the most negatively rated practice suggests that leaders need to use this practice, especially recognition and other informal methods, to trigger their subordinate’s motivation, as indicated by Sankar et al. (1991). This fact is supported by a study of 26 project teams in different industry sectors, namely chemical, technology, and consumer products, in which rewarding team members upon achievement of goals was observed as one of the critical behaviors of a leader that has a positive impact on facilitating team creativity and ultimately innovation (Amabile et al., 2004).

### Support to nurture creativity and innovative spirit provided to an individual and the team

The last set of nine statements relates to creativity and innovation. In this set, responses ranged from “not at all” (1) to “a very great extent” (6; Table 1). Table 1 shows that the lowest mean (3.88) relates to the ability of the manager to prepare for change and integrate new ideas and information, which could be because tenured scientists, those that generally have managerial positions, are holding onto their roles in their 60s and beyond. This might negatively impact innovation as several new ideas might be rejected. On the other hand, the question regarding “the level of autonomy offered” recorded the highest mean with a value of 4.56, although the delegation level was rated low. This suggests that subordinates might be left working autonomously on projects rather than working on tasks to help their managers, which could further empower them.

For this section, all statements except those relating to “team adaptation to change and idea integration” and “nurturing of openness and opportunities” recorded between 50 and 67% of positive feelings. The maximum negative emotion was recorded for a question relating to “nurturing of openness and opportunities” with 20.3%, which is in correlation with the relatively low positive feeling expressed for “Developing and mentoring” (statement 25; 40.6%) and “Networking” (statement 28; 35.9%). Taken together, this suggests that managers might hinder the possibility of their collaborators learning more and possibly evolving to new horizons. Besides, it has been shown that leadership actions directed towards coaching, developing, and mentoring the team promote team progressivity and effectiveness (Hackman and Wageman, 2005), positively impacting creativity and innovation. Findings from the training and development functions of four team leaders across 14 teams in a Swedish manufacturing plant showed that the team leaders were proactively in the team’s task and engaged in developing team members’ knowledge, skills, and team spirit. Further, the role of a leader in mentoring, training, and development was positively related to team innovation and creativity (Dackert et al., 2004). This study indicates that even though the academic environment provides the opportunity for scholars to nurture their talent, this opportunity is inadequate without support, mentoring, and developing leadership functions.

## Discussion

To gain insights into the effects of the behavioral traits of leaders display on team creativity and innovation within the R&D environment, we need a better understanding of the team’s views on leadership styles and motivational support to enable creativity and innovation in the academic environment. In the R&D environment, particularly the academic, cross-functional teams are observed more regularly and, when properly managed, can positively affect team creativity and innovation. By providing empirical evidence on the views on leadership styles and functions of team members of academic-based teams in the R&D environment, this research provides a richer insight into how teams and their subordinates can be engaged and motivated to nurture and thus possibly promote creative thinking and hence innovativeness. Therefore, a questionnaire was used to explore these relationships and target the R&D environment, particularly the academic environment, to gain insights into how leadership influences motivation, creativity, and innovation.

### Demographics data outcome

A total of 86% of the participants in this study were employed in the academic sector, setting a good ground for the study, as the aim of the study was to look at the R&D environment and preferably academia. The study observes that a Ph.D. level is necessary for academia to reach higher positions, such as middle and higher-range manager positions. However, gender inequality was noticed as fewer females were in senior professional positions. This is in agreement with previous studies (Shen, 2013), which showed that women do not have equal chances to reach high-profile positions. However, the correlation analysis revealed that the gender itself played a limited influence on the outcome of the results.

### Personal work perception of individual participants

The present study reveals that the participants were motivated by their work and liked their working environment. Over 70% of the participants were positive about the necessity for creativity and having the characteristics of innovation and creativity. Most importantly, none of the participants rated negatively about this later statement. It is intriguing to note that the participants have the necessary tools to perform their respective work but felt there was limited support among colleagues, depicting limited teamwork. The lack of teamwork was further emphasized in the correlation analysis that revealed that managers in academia tend to focus on individual performance and contribution. This can translate to high competitiveness in the academic environment that hinders the drive for collaboration, effective teamwork (Carson et al. 2018), cognitive flexibility, problem-solving, and creativity (Gagné and Deci, 2005). Previous studies have proposed that inadequacy in teamwork might impair commitments to projects and reduce exposure to novel ideas. Thus, this may reduce motivation and creative thinking (Abra, 1994; Cagliano et al., 2000). Therefore, academic leaders must focus on developing teamwork, collaborative work, and team spirit to benefit creativity and innovation. The present study also suggests that managers in an academic environment should focus on reducing the influence of group conflicts as a means to enhance creativity and innovative thinking. Furthermore, in a knowledge-based economy, creativity and innovation capacity are essential elements. Therefore, the individual’s innovation success depends on an employee’s relationship with the organization that provides information, resources, support, and inspiration that helps innovators to promote and develop new ideas.

### Effect of the leader’s behavior and management style on subordinates

Of the Managerial Practices Categories, “Informing,” “Planning and Organizing,” “Supporting,” and “Clarifying roles and objectives” showed a positive relationship with leadership in the R&D environment. This observation suggests that leaders in R&D environments provide subordinates with a clear plan and organization and that they can communicate effectively and efficiently. The root of success in a team is fostered through leadership that provides adequate support and planning and is likely to be beneficial and facilitate the completion of tasks in addition to creative and innovative thinking. Appreciation of the working environment is also expected to trigger motivation and inspiration. It is, therefore, essential for managers to work on improving the work environment as a way to foster extrinsic motivation of their subordinates, notably when this fosters the feeling of autonomy and competence in their subordinates, as described in the cognitive evaluation theory (Ryan, 1982).

### Support to nurture creativity and innovative spirit provided to an individual and the team

Results showed that managers in academic environment offer autonomy to their subordinates, which can provide intrinsic motivation and drive performance, notably creativity and innovation (Gagné and Deci, 2005). This positively offers self-management and the opportunity for the subordinates to rely more on their resources and become more resilient and adaptable. However, this also means they must be proactive and autodidact, skills not developed by everyone, especially young professionals or students. Thus, managers must focus on adapting their managerial styles to subordinates, notably by developing emotional intelligence (Moss et al., 2006).

Leadership functions involving consulting, delegation, and rewarding had negative feedback in this study. These three negatively rated practices suggest a limitation in celebrating success in R&D compared to other industrial environments, as previously reported (Amabile et al., 2004). Additionally, the analysis of statements 31–39 revealed that leaders in academic environments have a limited ability to embrace change and integrate new ideas, possibly because higher manager positions are obtained by highly experienced and, thus, aging leaders. Overall, this is likely to have a negative impact on innovation and creativity. Taken together, it appears that leaders in academia allow their subordinates to work autonomously rather than delegating and empowering them, limiting the subordinates’ opportunities to evolve to new horizons and higher positions. Interestingly, this might reinforce the high competitiveness observed in academia and described in the analysis of the demographic data. Dackert et al. (2004) stated that mentoring was positively related to team innovation and creativity. This, in turn, suggests that leaders should emphasize developing support and mentoring. Worth noting is that leaders in academic environments seem to offer little rewards, indicating the limited opportunities they provide to their subordinates.

## Conclusion and recommendations

The present research intended to answer two research questions with the aim of determining if the leader’s behaviors influence subordinate creativity and innovation in academic R&D environments. On the contrary, the relationship between the production of ideas and the leadership role in fostering team innovation and creativity is examined. The first research question that the study disclosed was to what extent the “PMEG framework” impacts leadership in an R&D environment. In this regard, the positive effect is that group interactions lead to fostering team creativity and innovation. It thus appears that “people” influence logical variance between followers and leaders, “means” carry activities to influence followers, “effects” include certain heterogeneous reactions to followers, and finally, “goals” are essential for directing innovation. Thus, these four dimensions allow a systematic framework for making leadership easier and positively impact leadership (explained in the above sections) that influences an individual’s attitudes and behaviors and interaction between groups.

The second research question that the study intended to answer was to understand the production of ideas in an organization as well as the relationship between the creation of ideas and leadership roles. Therefore, the results show that the relationship between them can be positive, as reflected in the literature that managers of the academic R&D environment can improve to foster their subordinates’ motivation to trigger creative thinking and innovation. Thus, every organization should nurture innovation appropriately and particularly to stay competitive. Finally, it reveals that innovation does not take place without change. We have put the above hypotheses for testing, and the results are discussed in our study. The survey’s findings revealed that managers display behaviors that influence creativity and innovation, some in a positive way, which can enhance creativity and innovation, but also some that can negatively impact creativity and innovation. The positive behaviors included providing adequate support, planning, organization, and communicating effectively and efficiently. The survey also revealed that managers in the academic R&D environment focus on reducing the influence of group conflicts. On the other hand, the survey’s data also showed that managers tend to focus on individual performance and contribution.

The findings also had applications for managers hoping to foster creativity among their employees. First, managers should utilize transformational leadership primarily to develop a person’s creative instinct. Additionally, managers have the potential to be transformative leaders. High-caliber managers should have the appropriate training to serve as role models for their staff. Second, management should hire leaders who provide each employee the specific attention they need, in addition to training technocrats. Such an event encourages staff creativity and increases employee motivation. Thirdly, managers or supervisors need to accurately translate their ideals into concrete objectives so that staff members can work to achieve the targets. Additionally, transformational leaders should provide their team members the freedom to experiment with new concepts, look for intellectually stimulating challenges, and foster their creativity.

Along with making a significant contribution, the study has limitations that must be taken into account while explaining its results. By conducting a cross-regional and cross-cultural study, researchers could better understand how ethical leadership impacts employee and corporate outcomes. Further investigation is needed on the moral conduct of managers in service-related industries, such as hospitals, where nurses and doctors may influence their staff members’ behavior to foster creativity or innovation. This study also demonstrates that for innovation to be implemented in firms, executives must be inspired to welcome employee ideas.

### Ideas for future research

Although the present study offers theoretical knowledge and insights about the processes that can trigger and impair creativity and innovation in academic R&D environments. Notably, it would be insightful to conduct a survey focusing on subordinates only to differentiate data gathered between subordinates and managers. In the present study, the entire data set was analyzed rather than separated according to the position held among participants. A follow-up analysis based on semi-structured interviews would also allow for deepening the knowledge and help understand better the meaning of answers gathered in the survey. For example, interviews could bring a better understanding of the feelings behind the development of autonomy, role clarity, and the work environment in academic environments and help to understand the intricate roles of these towards creativity and innovation. The data collected from interviews would, however, need to be analyzed using a qualitative method. However, Rahman (2016) states that there are certain disadvantages to the quantitative research approach. Firstly, using this research method requires us to be prepared financially. Also, as we need an enormous number of respondents, we need cash for questionnaire printing, transportation fees, etc. Secondly, positivism cannot address how social reality is retained and shaped or how people interpret their actions. Thirdly, this research method requires a larger sample of people, leading to more statistical accuracy.

On the other hand, Rahman (2016) claims that this research method overlooks the respondents’ experience because when collecting data, there seems to be an indirect connection between the researchers and the respondents. Lastly, it proclaims another limitation of this research method is that it is inclined to take a screenshot of a phenomenon, measure variables at a specific moment in time, and disregard whether the photograph looks unusually disarranged. It has been reported that integrating quantitative and qualitative data analysis multiplies the potential and likelihood of unanticipated outcomes, meaning that the outcome gives new understandings and new insights (Bryman, 2006). Besides, a perception of a multidisciplinary approach can be tested to validate whether leaders in academia promote trans-disciplinary research and the influence this has on creativity and innovation as well as on the expansion of collaborative work. Finally, the authors have also highlighted that few academic contributions have explored the role of leadership in an R&D environment. In this perspective, more research needs to be carried out on the dynamics of the R&D environment to foster team innovation and creativity.

## Data availability statement

The raw data supporting the conclusions of this article will be made available by the authors, without undue reservation.

## Ethics statement

The studies involving human participants were reviewed and approved by University of Roehampton Online. The ethics committee waived the requirement of written informed consent for participation.

## Author contributions

SS contributed to conception and design of the study. SA organized the database. LT and ZH performed the statistical analysis. ZH, SS, LT, and SA contributed to writing the first draft of the manuscript. All authors contributed to the article and approved the submitted version.

## Conflict of interest

The authors declare that the research was conducted in the absence of any commercial or financial relationships that could be construed as a potential conflict of interest.

## Publisher’s note

All claims expressed in this article are solely those of the authors and do not necessarily represent those of their affiliated organizations, or those of the publisher, the editors and the reviewers. Any product that may be evaluated in this article, or claim that may be made by its manufacturer, is not guaranteed or endorsed by the publisher.
